# Clp ATPases differentially affect natural competence development in *Streptococcus mutans*

**DOI:** 10.1002/mbo3.1288

**Published:** 2022-05-15

**Authors:** Satya D. Pandey, Indranil Biswas

**Affiliations:** ^1^ Department of Microbiology University of Kansas Medical Center Kansas City Kansas USA

**Keywords:** Clp ATPase, growth phase, lactic acid bacteria, microbial development, natural competence

## Abstract

In naturally competent bacteria, DNA transformation through horizontal gene transfer is an evolutionary mechanism to receive extracellular DNA. Bacteria need to maintain a state of competence to accept foreign DNA, and this is an energy‐driven phenomenon that is tightly controlled. In Streptococcus, competence development is a complex process that is not fully understood. In this study, we used *Streptococcus mutans*, an oral bacterium, to determine how cell density affects competence development. We found that in *S. mutans* the transformation efficiency is maximum when the transforming DNA was added at low cell density and incubated for 2.5 h before selecting for transformants. We also found that *S. mutans* cells remain competent until the mid‐logarithmic phase, after which the competence decreases drastically. Surprisingly, we observed that individual components of Clp proteolytic complexes differentially regulate competence. If the transformation is carried out at the early growth phase, both ClpP protease and ClpX ATPase are needed for competence. In contrast, we found that both ClpC and ClpE negatively affect competence. We also found that if the transformation is carried out at the mid‐logarithmic growth phase ClpX is still required for competence, but ClpP negatively affects competence. While the exact reason for this differential effect of ClpP and ClpX on transformation is currently unknown, we found that both ClpC and ClpE have a negative effect on transformation, which was not reported before.

## Graphical abstract

Natural competence development in bacteria is modulated by several factors including environmental conditions, availability of nutrients, and growth conditions. *Streptococcus mutans*, an oral bacterium and the causative agent of dental caries, develops natural competence. The process of competence development in this bacterium is not fully understood. Here, we observed that *S. mutants* remains competent for several hours, which is in contrast to other streptococci. We also observed that several proteolytic complexes encoded by Clp proteins, which are generally needed for proper maintenance of cellular proteome, differentially regulate competence development in this oral streptococcal species. This is somewhat surprising since Clp proteolytic complexes are known to regulate competence development only negatively. These novel findings will potentially open a new avenue of research toward understanding the regulation of competence development in streptococci.

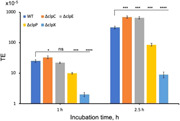

## 1 INTRODUCTION

Diverse phyla of both Gram‐positive and Gram‐negative bacteria develop a special physiological state that allows them to uptake DNA from the environment. This state, which is commonly known as natural competence, is generally transient and develops at different stages of growth depending on the bacterial species. Natural competence development has been thoroughly studied in a few Gram‐positive and Gram‐negative bacterial species (Blokesch, 2016; Dubnau, 1991; Dubnau & Blokesch, 2019; Johnston et al., 2014). In *Bacillus subtilis*, the natural competence is developed when the cells enter the stationary growth phase and the cells retain competence for a short time (Dubnau, 1991). On the other hand, in *Streptococcus pneumoniae*, competence development occurs in a cell density‐dependent manner, which occurs in the early to mid‐log growth phases (Salvadori et al., 2019; Tortosa & Dubnau, 1999; Waters & Bassler, 2005). In some organisms such as *Neisseria gonorrhoeae* and *Helicobacter pylori*, the cells are constitutively competent throughout the growth stages (Aas et al., 2002; Dubnau & Blokesch, 2019; Hamilton & Dillard, 2006; Hofreuter et al., 2000). It is thought that nutritional status and environmental stresses can modulate competence development (Aminov, 2011; Finkel & Kolter, 2001). Furthermore, induction of competence also depends on certain biopolymers such as chitin, as is the case for *Vibrio cholerae* (Blokesch, 2016; Matthey & Blokesch, 2016; Seitz & Blokesch, 2013; Stutzmann & Blokesch, 2020).

Streptococci are a diverse group of organisms that are represented by over 100 different species (Andam & Hanage, 2015; Facklam, 2002). Among the streptococci, a handful of species have been characterized for natural competence development (Johnston et al., 2014; Salvadori et al., 2019). Our knowledge of natural competence development in streptococci mostly comes from studies on *S. pneumoniae* (Claverys et al., 1997; Straume et al., 2015). In this bacterium, competence is induced by a short 17‐residue unmodified peptide pheromone called a competence‐stimulating peptide (CSP). This CSP is secreted by a dedicated ABC transporter that cleaves off a leader peptide during transport from the 45‐residue pre‐CSP encoded by the *comC* gene. Similarly, in *S. mutans*, 21‐residue pre‐CSP (CSP‐21) peptide undergoes postexport processing at the C‐terminal end that creates a relatively more potent 18‐residue peptide (CSP‐18) that works at a much lower concentration than CSP‐21 (Petersen et al., 2006). When the extracellular concentration of CSP reaches a critical concentration, a membrane‐bound sensor kinase, ComD, senses the signal and is activated by autophosphorylation (Johnsborg & Havarstein, 2009; Martin et al., 2010). The phosphorylated ComD then transfers the phosphate group to its cognate cytoplasmic response regulator ComE for activation. ComD, ComE, and ComC are encoded by the *comCDE* operon (Martin et al., 2010). The activated ComE then induces the expression of a competence‐specific master sigma factor, *comX*, and the *comCDE* operon (Martin et al., 2013). Once ComX is made in the cell, it induces the expression of so‐called late competence genes that are needed for DNA processing, uptake, and recombination (Luo et al., 2003; Martin et al., 2013; Morrison & Lee, 2000). Typically, *S. pneumoniae* cultures develop competence in the mid‐logarithmic phase and last nearly 30 min (Alloing et al., 1998). Exogenous addition of CSP leads to induction of *comX* transcription within 5 min and the transcript disappears by 15 min indicating why competence is short‐lived (Alloing et al., 1998). Furthermore, the ComX protein is also degraded rapidly by the ClpC/P and ClpE/P proteolytic complexes that finally cease the competence (Chastanet et al., 2001; Weng et al., 2013).

In streptococci, ComX is the master regulator of competence development since it directly controls the expression of the late competence genes (Claverys & Martin, 1998). However, the expression of *comX* is not always dependent on the ComD/E two‐component system (TCS). In certain streptococci, including *Streptococcus thermophilus*, a second signal transduction system called ComR/S is also involved in *comX* activation (Fontaine et al., 2015). In this case, *comS* encode a short peptide that is secreted outside the cell as an 11‐residue long pheromone called ComX‐inducing peptide (XIP) (Federle & Morrison, 2012; Mashburn‐Warren et al., 2010). When the XIP reaches a certain density, it is imported back into the cell through OppA, an oligo‐permease. XIP then interacts with the ComR transcriptional regulator and stimulates binding the ComR‐XIP complex to the promoter of *comX* and *comS* and induces transcription. This ComR/S dependent competence induction is only seen in chemically defined media (CDM) that is devoid of oligopeptides, and in this medium, *S. thermophilus* becomes spontaneously competent (Fontaine et al., 2015). The induction of early *com* genes in *S. thermophilus* is similar to *S. pneumoniae* and occurs in the early‐ to mid‐log phase (at low cell density). However, in contrast to *S. pneumoniae* and *S. thermophilus*, in *Streptococcus infantarius*, competence develops at the late log phase (high cell density) and lasts for a short time (Danne et al., 2013). *S. infantarius* competence development is dependent on the ComR/S pathway and not the ComD/E. Despite the presence of all the genes necessary for natural competence development, some streptococci never develop competence (e.g., *Streptococcus gallolyticus*), while some others, such as *S. pyogenes*, develop competence only under specific conditions, such as in biofilm (Danne et al., 2013; Marks et al., 2014).

*Streptococcus mutans* is an oral pathogen associated with dental caries formation and often causes endocarditis. This organism develops competence at the late log phase that persists several hours after induction (Desai et al., 2012). Interestingly, in *S. mutans* both ComD/E and ComR/S sensor systems modulate the expression of *comX* depending on the growth media (Khan et al., 2016; Reck et al., 2015; Underhill et al., 2018). In nutrient‐rich media, ComD/E appears to play a major role, and competence is induced when exogenous CSP is added. Deletion of the ComDE pathway does not abolish competence but transformability is reduced by several orders of magnitude. On the other hand, the ComR/S system is necessary for competence development in CDM. Deletion of ComR also leads to complete cessation of competence development irrespective of the growth media. In *S. mutans*, the ClpC/P complex was shown to be negatively involved in competence by altering the ComX protein concentration in the cell. However, the effect of ClpC/P was only observed in nutrient‐rich medium and not in CDM (Dong et al., 2014; Tian et al., 2013). *S. mutans* regulates genetic competence through multiple layers of control. Working on *S. mutans*, Son and colleagues (2018) show that posttranslational control of ComX by MecA/ClpCP is active under both XIP and CSP stimulation. The MecA/ClpCP blocks adventitious entry into competence by sequestering or intercepting low levels of ComX but permits the competence only when ComX levels exceed a threshold. However, cell‐to‐cell heterogeneity in MecA levels creates variability in that threshold. Therefore, MecA/ClpCP provides a stochastic switch, located downstream of the already noisy *comX*. In *S. mutans*, a third signal transduction system encoded by HdrRM appears to modulate competence in a cell density‐dependent manner (Okinaga et al., 2010). The exact mechanism by which HdrRM exerts its effect is currently unknown.

The main goal of this study was to determine the optimum environmental conditions to obtain maximum transformation efficiency (TE) under nutritionally rich conditions. Our results indicate that maximum transformation occurs near mid‐exponential growth phases and the cells remain competent for a long time. We found that the ClpP protease drastically affects competence in a growth phase‐dependent manner. In the early stages of growth, both ClpX ATPase and ClpP positively affect competence, while at a later growth stage, ClpP negatively affects competence while ClpX has a negligible effect.

## 2 MATERIALS AND METHODS

### 2.1 Strains, media, and growth conditions

*S. mutans* UA159 and its isogenic mutants Δ*clpX*, Δ*clpE*, Δ*clpP*, and Δ*clpX* (Tao & Biswas, 2015), as well as GS‐5, were routinely grown in Bacto Brain Heart Infusion (BHI; Becton Dickinson) at 37°C under microaerophilic conditions (candle jars), in the presence or absence of antibiotics, such as erythromycin (Em; 1 or 10 μg/ml). DNase I (Sigma) was added when needed as described in the text.

### 2.2 Optimization of transformation assay for natural competence

**Figure 1:**
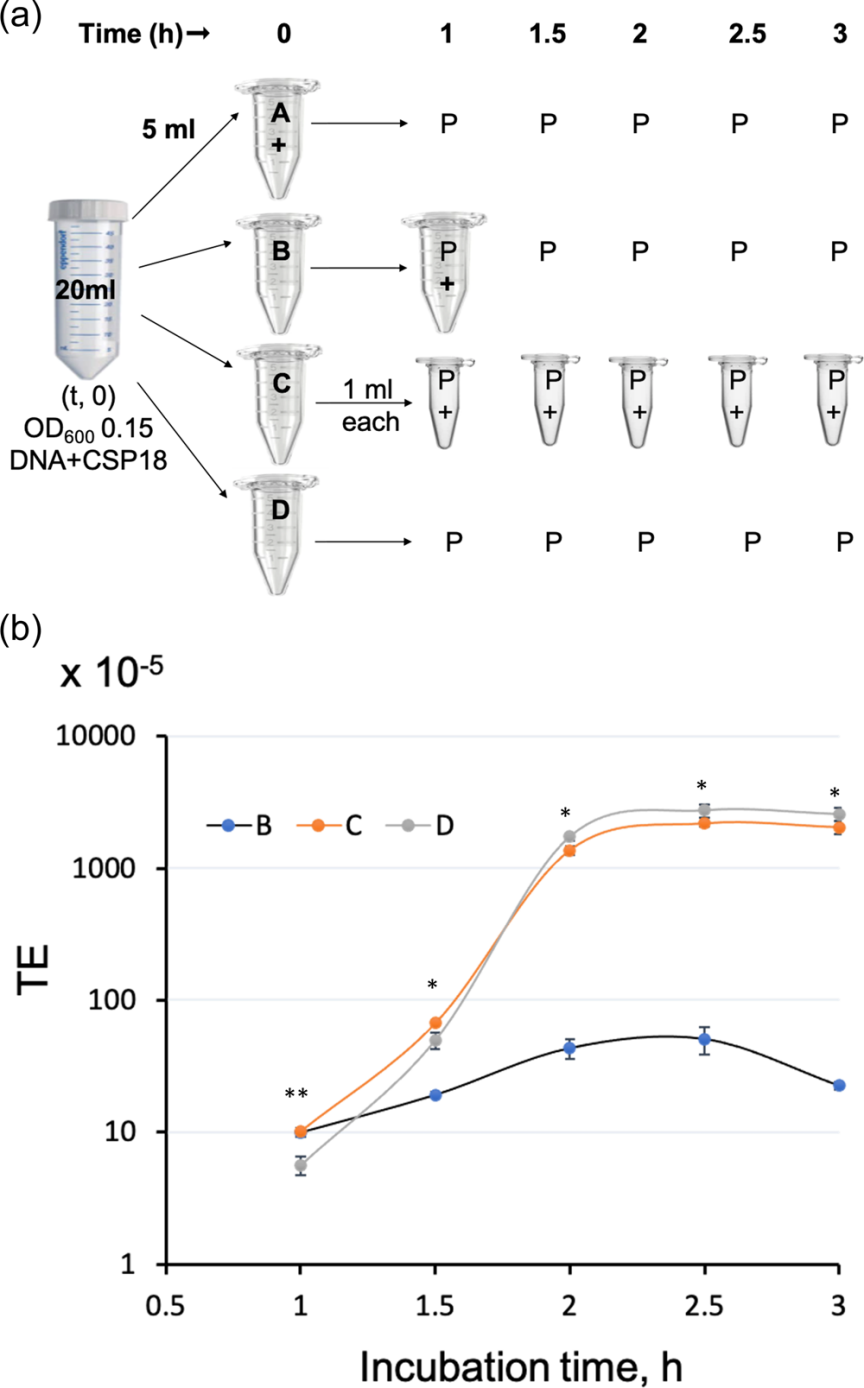
Experimental strategy to evaluate transformation efficiency in UA159. (a) Schematic representation of the strategy to evaluate DNA uptake using DNase I (+). At culture OD_600_ = 0.15 (considered time zero; *t*, 0), DNA (pIB184Em; 1 µg/ml) and CSP18 (200 nM) were added, and the culture was equally distributed (5 ml each) in four groups (A–D). In Group A, DNase I (10 U) was added immediately (*t*, 0). In Group B, DNase I was added 15 min before plating (P) at 1 h followed by plating at different time points. In Group C, culture was further distributed equally in five vials (1 ml each), and DNase I was added into each vial at different time points just 15 min before plating. In Group D, no DNase I was added. Plating of an aliquot from the four groups was done at 1, 1.5, 2, 2.5, and 3 h of growth at 37°C. (b) Transformation efficiency plot of four different groups (a–d). The *y *axis is represented on the log10 scale and Group A was not shown in the graph due to the “zero” value. The values represent the “mean ± SD“ of three independent replicates. Statistical significance (*) between Groups C and D was calculated using a *t‐*test.

Strain UA159 was streaked on a BHI‐agar plate from glycerol stock (−80°C). The next day, a single colony was inoculated into BHI broth and allowed to grow overnight. The culture was diluted to 1:20 in BHI media containing 5% (v/v) horse serum (heat‐inactivated; Sigma) and allowed to grow up to OD_600_ 0.15. Plasmid DNA (pIB184Em; [Hossain & Biswas, 2012]), 1 µg/ml and CSP18 (SGSLSTFFRLFNRSFTQA), 200 nM were added at this point (time, 0) and divided in four equal volumes (Group A–D). In Group A, DNase I (10 U) was added at time 0, and an aliquot was plated at different time points. In Group B, culture was allowed to grow for 1 h and then DNase I (10 U) treatment was done for 15 min before plating. In Group C, after 1 h culture was further divided equally into five vials. DNase I was added in each vial separately at different time points for 15 min before plating. In Group D, DNase I was not added (Figure 1a). Plating from each group was done at 1, 1.5, 2, 2.5, and 3 h, and colonies were counted after 96 h. The TE was calculated as a percentage by dividing the number of transformants by the total number of colony‐forming units (CFU). To verify that the transformants are real and not spontaneous mutants, one colony each with and without antibiotic plates was grown in BHI‐media and genomic DNA isolated as described before (Hossain & Biswas, 2012). Using isolated genomic DNA as a template and gene‐specific primers in a polymerase chain reaction (PCR), the purity of the transformants was confirmed. The experiment was done in three replicates and values were presented as mean ± SD. The statistical significance was calculated using a *t‐*test and the *p* < 0.05 were considered significant.

### 2.3 Determination of TE at various OD_600 _point

The primary inoculum was prepared as above and cultures were grown in the presence of horse serum to OD_600_ 0.2. At this OD, CSP18 was added and cultures were divided equally into five vials. The DNA was added to each vial separately when the OD reached the desired density values (0.2, 0.35, 0.5, 0.65, and 0.8). A parallel set of cultures were also grown to determine the OD of the transforming cultures. Cultures were incubated for one more hour before plating. TE was calculated as described earlier. The experiment was done in triplicate and the values were present as mean ± SD. The statistical significance was calculated using a *t‐*test and the *p* < 0.05 were considered significant.

## 3 RESULTS

### 3.1 Early‐log transformation and late‐log plating yield higher TE

To better understand the optimum duration of incubation for DNA uptake by *S. mutans*, we used early‐log (OD_600_ = 0.15) grown culture. Transforming DNA (plasmid) and CSP18 were added at the same time. Our overall experimental strategy is shown in Figure 1a, where we equally divided culture into four groups, A–D. In Group A, we added DNase I immediately (time, 0), which served as control. As expected, we obtained no transformants on the antibiotic selection plate even after prolonged incubation (3 h). The data suggest that DNase I is highly effective in digesting DNA in the BHI medium. The absence of DNA was later confirmed by PCR as well (data not shown). In Group B, we added DNase I 1 ‐h postaddition of DNA and CSP18 followed by further incubation for different periods of up to 3 h. The idea was to assay the multiplicity of the transformed cells in the first hour of incubation. We observed a fivefold increase in transformants at 2.5 h when compared to 1 h (TE; 50.5 × 10^−5 ^vs. 10 × 10^−5^) before it started to decline. We believe this fivefold increase in the transformants is due to the multiplication of the transformed cells in the first hour of incubation. In Group C, we added DNase I at five different time points (five separate aliquots) at the interval of 30 min. This allowed the cells to adsorb and uptake the DNA for different lengths of time. As shown in Figure 1b, we observed a dramatic increase in TE as the incubation time progressed. After 2.5 h incubation, we obtained a nearly 218‐fold increase in transformation compared with 1‐h incubation (TE; 2181 × 10^−5 ^vs. 10.2 × 10^−5^). This 218‐fold increase in Group C compared to just fivefold in Group B at 2.5 h suggests that length of incubation time plays an important role in overall transformation. In Group D, we did not add DNase I and as expected, TE was the highest; nearly 488‐fold observed at 2.5 h when compared to the first hour (TE; 2.73 × 10^−2 ^vs. 5.6 × 10^−5^) in Group D.

When we compared the TE between Groups C and D, we observed a higher efficiency in Group D than in Group C at the longer incubation times such as 2, 2.5, and 3 h. However, surprisingly at the early growth stages (1 and 1.5 h), we noted an increase of 2‐ and 1.4‐ fold in the efficiency in Group C (Figure 1b). Since this result was consistent we hypothesize that sheared plasmid in Group C could have transformed quicker than the full‐length plasmid in D and circularized inside the host. To test the hypothesis, we partially treated the transforming plasmid DNA with 0.1 U of DNase I for 1 min or linearized the plasmid by restriction digestion with *Xho*I and used it for transformation. We observed no transformant on the selective plate when partially sheared or linear plasmid was used for transformation (data not shown). We also noticed that there was a more viable number of colonies in Group A at early growth stages compared to other groups (nearly twofold). The data suggest that the addition of DNase I in growth media (BHI) somehow enhanced the bacterial growth that could have resulted in higher TE in the early growth stage in Group C. Overall, we observed that maximum transformation is obtained when the transformation is carried out at OD_600_ 0.15 and incubated for 2.5 h.

### 3.2 Low cell density transformation yields higher TE

**Figure 2:**
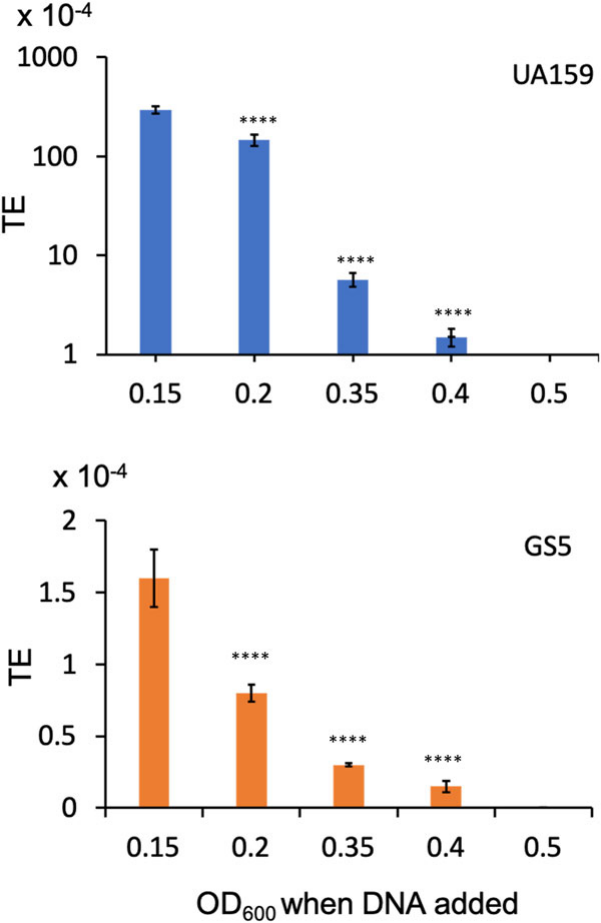
Comparison of transformation efficiency of two *Streptococcus mutans *strains. Transforming DNA (plasmid) and CSP18 were added at indicated culture densities and incubated for 2.5 h before plating on selective plates. The transformation efficiency values of the two strains are shown with the “mean ± SD” of three independent replicates. Statistical significance (*) was calculated using one‐way analysis of variance and values were compared against OD 0.15 in both the panels.

To understand the role of culture density on transformation, we added DNA and CSP18 at different OD_600_ points (0.15, 0.2, 0.35, 0.4, and 0.5) and incubated them further for 2.5 h before plating. The reason was to induce competence at the time of DNA addition and not before that. As shown in Figure 2, the maximum transformation was obtained at the earliest time point (OD_600_ = 0.15). The transformation was dramatically decreased as the culture density was increased. We obtained no transformant when DNA was added at the mid‐log phase (OD_600_ = 0.5). To further confirm these results in another strain of *S. mutans*, we used GS5. We found that overall TE was nearly 180‐fold less in GS5. However, we also observed that transformation was highest at the earliest time point (OD_600_ = 0.15) and then gradually decreased as the cell density increased, a pattern similar to what was observed with UA159. Taken together, the results suggest that the competence development in UA159 and GS5 starts at very low cell density (OD_600_ = 0.15) and continues throughout the growth stages, though with a gradually lower rate, up to the mid‐log phase (OD_600_ = 0.5) before reducing abruptly. And therefore, the optimal time of incubation, 2.5 h, was necessary to attain overall a maximum TE.

**Figure 3:**
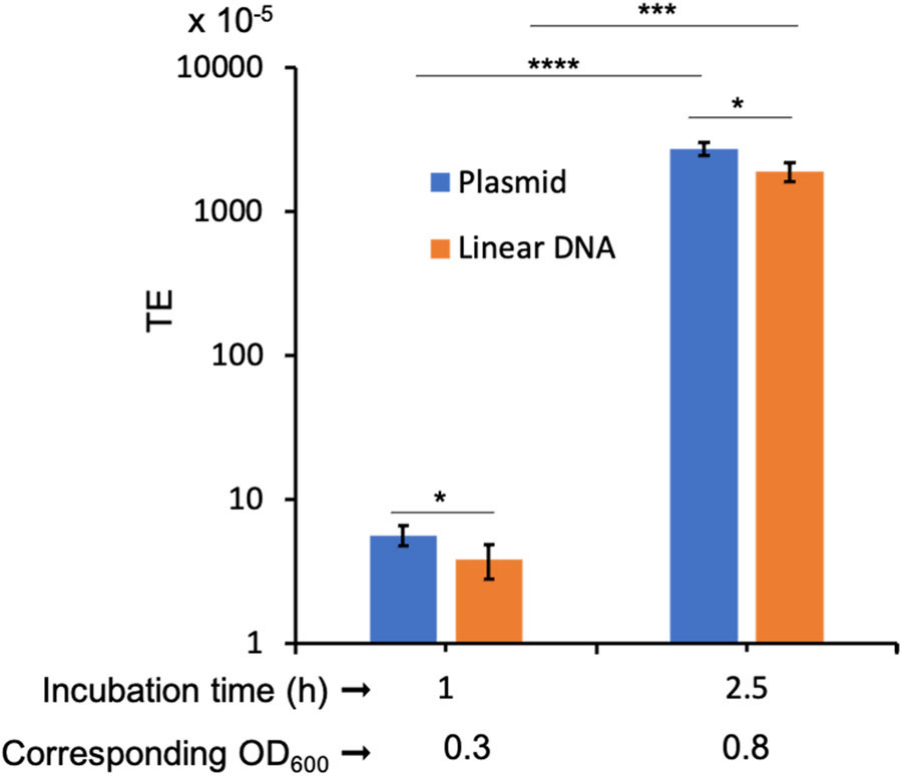
Transformation of circular and linear DNA molecules. Transforming DNA (plasmid or linear) and CSP18 were added at OD_600_ = 0.15 and the culture was further incubated for 1 or 2.5 h, as indicated. OD_600_ of the culture was measured at the time of plating. The values represent the “mean ± SD“ of three replicates. Asterisk (*) shows significant *p* values (<0.05) calculated using a *t‐*test.

The exact mechanism by which plasmid DNA is taken up by the competent cells is poorly understood. It is thought that at least two copies of plasmid DNA need to be simultaneously taken up by the cell to recircularize inside the cell to generate a complete functional plasmid molecule (Barany & Tomasz, 1980; Lacks, 1979; Saunders & Guild, 1981). Thus, the transformation conditions described above may be specifically suitable for plasmid DNA. To rule out the possibility, we transformed a linear DNA that contains an *ermA* gene (same resistance gene carried on the plasmid) that is flanked by ~500‐bp homology to the SMU.198 locus; Hossain & Biswas, 2012) and compared it with the plasmid DNA transformation. We added DNA at OD_600_ 0.15 and incubated for 1 and 2.5 h. As shown in Figure 3, we obtained maximum transformation after 2.5 h incubation. To our surprise, we also found that the TE of plasmid DNA was ~1.5‐fold more as compared to the linear DNA at both incubation time points (Figure 3).

### 3.3 The *clpP* deletion mutant showed higher TE than WT and the *clpX* deletion mutant when transformed at a higher cell density

**Figure 4:**
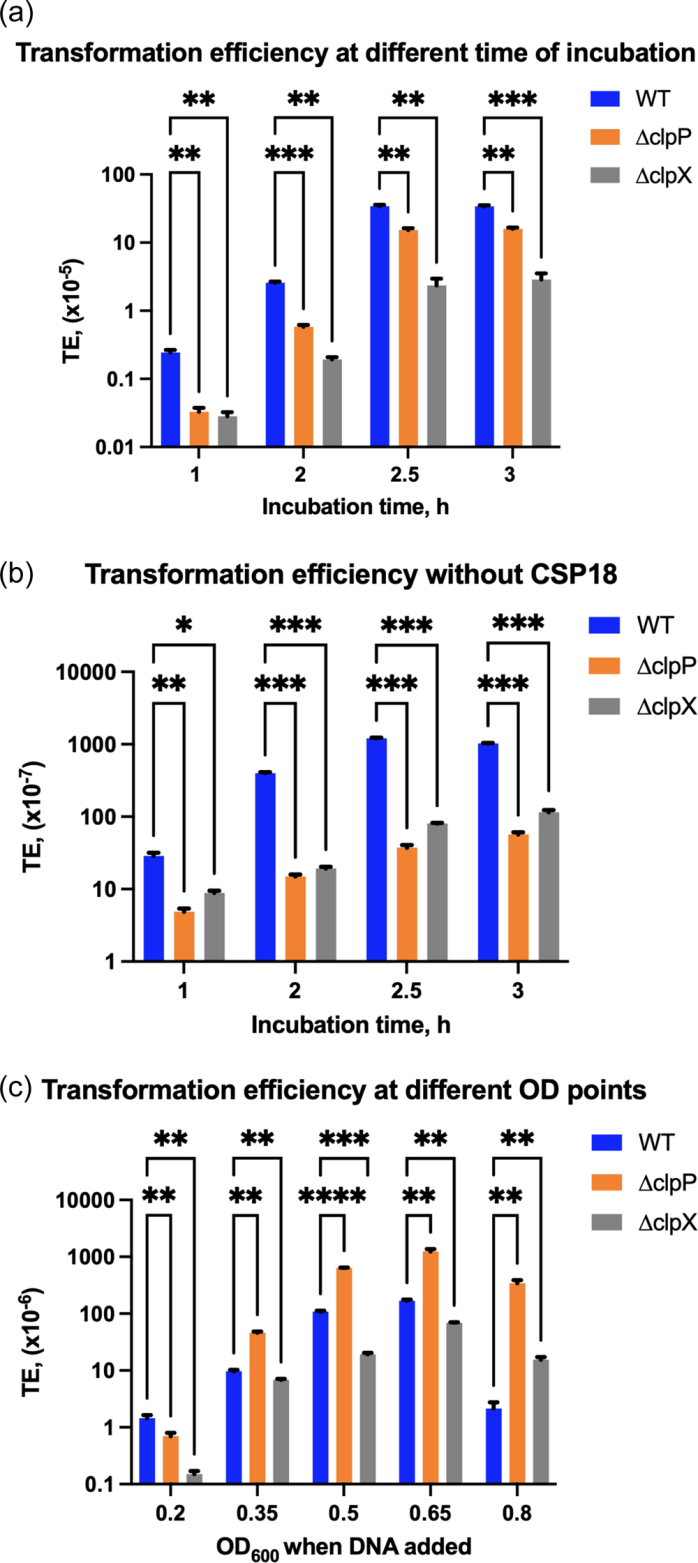
Effect of Clp proteins on transformation. (a) Transformation efficiency of UA159 (wild type) and its isogenic *clpX* and *clpP* mutant strains. Transforming DNA (plasmid) and CSP18 were added at culture density OD_600_ = 0.15, and the culture was further incubated for indicated time before plating on selective plates. (b) A parallel experiment similar to (a) except CSP18 was not added into the culture. (c) Transforming DNA (plasmid) was added at various culture densities as indicated (OD_600_ points between 0.2 and 0.8) followed by 1 h incubation before plating. Asterisk (*) shows a significant *p* value (<0.05) in a particular time point calculated using the *t‐*test.

Transformation is dependent on the activity of Clp proteolytic complexes in streptococci and other bacteria (Dong et al., 2014; Fontaine et al., 2015; Son et al., 2018; Wahl et al., 2014). Others and we have previously shown that ClpP affects transformation in *S. mutans* (Chattoraj et al., 2010; Lemos & Burne, 2002). ClpP forms an active proteolytic complex by association with an ATPase and in *S. mutans*, the major Clp proteolytic complex is ClpXP (Kajfasz et al., 2009, 2011). To study the role of ClpP and ClpX in transformation, we used isogenic Δ*clpX* and Δ*clpP* mutants of the UA159 strain (Jana et al., 2016; Tao & Biswas, 2015). Both these mutants are clean‐deletion without any antibiotic resistance markers (Tao & Biswas, 2015). We then evaluated the TE of the strains by adding CSP18 and DNA at low cell density (OD_600_ = 0.15), and without CSP18. We observed a reduction in TE of up to two to three orders of magnitude in absence of CSP18 in all the three strains during the incubation up to 3 h. We found that both Δ*clpX* and Δ*clpP* strains displayed a defect in transformation under both conditions (with and without CSP18) when compared to the wild‐type (WT) UA159. Interestingly, we observed a higher rate of TE in Δ*clpP* than Δ*clpX* in culture treated with CSP18; however, an opposite trend was observed in culture without CSP18 treatment (Figure 4a,b). At 2.5 h incubation in culture with CSP18, both the Δ*clpP* and Δ*clpX* strains showed about 2.2‐ and 15‐fold reduction in TE, respectively, as compared to the WT. The fold difference for the Δ*clpP* was slightly reduced (~2‐fold) after 3 h incubation.

**Figure A1:**
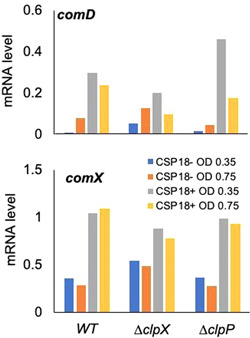
Transcriptional expression of *comD* and *comX* using semiquantitative polymerase chain reaction (PCR). RNA was isolated at two experimental conditions, without and with CSP18 (−/+), and OD 0.35 and 0.75. After removing gDNA contamination from the RNA samples, cDNA was prepared using company recommendations. Then PCR was done using gene‐specific primers. The band intensities of the PCR products were analyzed using ImageJ. The 16S primer was used as an internal positive control standard. Although the expression of *comX* in CSP18 treated cells was higher than the untreated ones for all three strains, their levels were close. However, the transcription of *comD* in the *clpP* mutant was dramatically higher than in the wild type (WT) and the *clpX* mutant. It could be a possibility why *clpP* mutant had higher transformation rates when DNA was added at between 0.35 and 0.65 (Figure 4c).

We then wanted to study whether the effect of Clp proteins is culture density‐dependent. To confirm, CSP18 was added at low cell‐density (OD_600_ = 0.15) to induce competence and the transforming DNA was added at different cell densities (OD_600_ = 0.2, 0.35, 0.5, 0.65, and 0.8) followed by 1 h incubation at 37°C. Surprisingly, we observed that Δ*clpP* strain generated higher transformation as compared to the WT UA159 at cell densities between OD_600_ = 0.35 and 0.8. For all the strains, TE reached the maximum at OD_600_ = 0.65. At this cell density, Δ*clpP* strain generated nearly eightfold more transformants compared to the WT. However, we observed a twofold lower transformation in the Δ*clpP* mutant than the WT UA159 at very low cell density (OD_600_ = 0.2). Afterward, it gradually increased and reached the maximum at OD_600_ = 0.65. In contrast to the Δ*clpP*, transformation efficiencies of the Δ*clpX* mutant were lower for all the cell densities when compared to the WT (Figure 4c). To gain insight into the mechanism, we measured the transcript of *comD* and *comX* by reverse transcription (RT)‐PCR (see Figure A1). To our surprise, we found that the expression of *comD* was higher in the *clpP* mutant as compared to the WT and the *clpX* mutant. The significance of this observation is not clear at this moment. Taken together, our observation suggests that ClpP and ClpX modulate transformation differently.

### 3.4 **Role of various** *clp* **proteases on TE of UA159**

**Figure 5:**
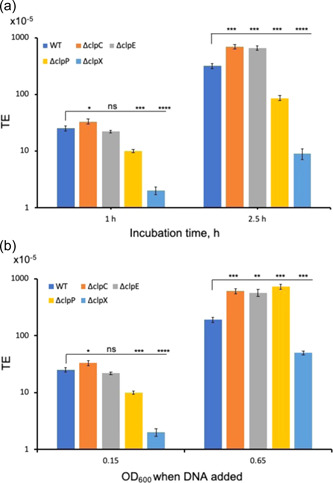
Effect of Clp ATPases on cell density. (a) Transformation efficiency at low cell density. Transforming DNA (plasmid) and CSP18 were added at culture density OD_600_ = 0.15, and the culture was further incubated for indicated time before plating on selective plates. (b) Transforming DNA (plasmid) was added at two culture densities as indicated **(**OD_600_ points at 0.15 and 0.65) followed by 1 h incubation before plating. CSP18 was added at OD_600_ = 0.15 for both the cultures. Asterisk (*) shows significant *p* values (<0.05) calculated using the *t‐*test. ns, not significant.

Since ClpP forms a proteolytic complex with two other ATPases, ClpC and ClpE, we wanted to verify whether these ATPases play any role in transformation. We added DNA and CSP18 at low cell density (OD_600_ = 0.15) and incubated the culture for 1 and 2.5 h (Figure 5a). In a parallel set of experiment, DNA was added at high cell density (OD_600_ = 0.65) to a CSP18 pretreated culture (at OD_600_ = 0.15) and incubated further for 1 h (Figure 5b). As expected, when DNA was added at low cell density (OD_600_ = 0.15) to the CSP18 pretreated culture, it produced very few transformants after 1 h incubation when compared to the 2.5 h incubation for all the isogenic strains. However, at 2.5 h of incubation, both the Δ*clpC* and Δ*clpE* strains produced nearly twice as many transformants as compared to the WT. As expected, the *ΔclpP* and *ΔclpX* strains produced nearly 3.5‐ and 35‐fold less transformants when compared to the WT (Figure 5a). On the other hand, when DNA was added at high cell density, both Δ*clpC* and Δ*clpE* strains produced nearly threefold more transformants as compared to the WT. Surprisingly, we found that the *ΔclpP* strain produced nearly fourfold more transformants than the WT. The TE remained low for the Δ*clpX* strain (fourfold lower than the WT) (Figure 5b). Taken together, our data indicate that the effect of ClpP protease on transformation is cell density‐dependent and different Clp ATPases have different effects on transformation.

## 4 DISCUSSION

In this study, we attempted to identify the optimum conditions for natural transformation in *S. mutans* with respect to growth in the rich medium. During this study, we have made several observations that suggest that competence development in *S. mutans* UA159 in the rich medium is rather complex. To avoid the unnecessary step of processing the CSP21 peptide by SepM and subsequent accumulation (Hossain & Biswas, 2012), we used the CSP18 peptide at the time of addition of our transforming DNA. We found that competence development in UA159 is greatly dependent on cell density and the maximum TE was obtained when DNA was added at very low cell density. We also noted that to obtain maximum transformants, it is necessary to incubate the culture for 2.5 h after the addition of CSP and DNA. If the cultures are plated after 1 h incubation, very few transformants are obtained (Figure 1b). This is in contrast to the competence development in *S. pneumoniae* where 1 h postincubation is sufficient (Mortier‐Barriere et al., 2019). Therefore, it appears that either the DNA uptake process or the postuptake processing, such as recombination, is rather slow in UA159.

We also found that UA159 can be transformed until the mid‐log phase if the competence is induced at low cell density (OD_600_ = 0.15) by CSP18. We obtained transformants until OD_600_ of 0.8, after which no transformants were obtained. Our result is consistent with an earlier study that also showed no transformants could be obtained after the cell density reaches OD_600_ of 1.1 (Li et al., 2016). However, the reduction of competence is very drastic during the growth phase. We noticed that for the UA159 strain, a change in OD_600_ from 0.2 to 0.35 resulted in nearly a 25‐fold reduction in transformation; while for the GS‐5 strain, the reduction was about threefold. Although the exact reason for this reduction is presently unknown, it is possible that the addition of CSP18 at only low cell density was able to induce the late competence genes necessary for the expression of DNA uptake machinery. Nevertheless, this particular assay does not provide any insight into how long the cells can uptake DNA once the competence is induced.

Generally, the transformation of linear DNA with homology to the chromosome is much more efficient than the transformation with plasmid DNA (Lopez et al., 1982; Mannarelli & Lacks, 1984). This is because during transformation double‐stranded circular plasmid DNA is converted to single‐stranded linear DNA during uptake (Barany & Tomasz, 1980; Lacks, 1979; Saunders & Guild, 1981). This linear DNA is later converted to circular DNA with the help of another transforming molecule. Thus, it is assumed that at least two copies of the plasmid need to be in the recipient cell to generate a complete plasmid molecule after transformation. In contrast, linear DNA with homology to chromosome needs to only integrate into the chromosome locus using homologous recombination. Thus, it was surprising to observe that the TE of linear DNA was less than that of plasmid DNA (Figure 3). While the exact reason for this difference is not clearly understood, we speculate that since the linear DNA needs to integrate into the chromosome by double‐crossover recombination, the observed difference could be due to a less efficient recombination process rather than due to DNA uptake. Further systematic experimentation with various chromosomal alleles and different plasmid molecules is needed to confirm the difference.

We noticed that the overall TE varies greatly between the UA159 and GS5 strains (Figure 2). Strain‐dependent variations in natural competence have been reported in *S. pneumoniae* (Li et al., 2016; Palmer et al., 2013) and other nonstreptococci including Vibrio and Neisseria (Duffin & Seifert, 2010; Simpson et al., 2019). However, most of the competence‐related studies in *S. mutans* have been done with UA159 only. The TE observed in GS5 was nearly two orders of magnitude lower than the UA159 strain (Figure 2). However, both the strains show a similar trend in TE with respect to cell density. The results are comparable with the *S. pneumoniae* study where the TE among the clinical isolates vary nearly four orders of magnitude (Palmer et al., 2013). It is worth mentioning that others and we have found that many *S. mutans* clinical isolates are either poorly or nontransformable. For example, a recent study by Palmer and colleagues found that about 50% of the clinical isolates (7 out of 15) were recalcitrant to natural transformation (Palmer et al., 2013). When we compared the genomes of UA159 and GS‐5, we did not find any known competence‐related genes that were missing or altered in GS‐5. At present, the exact mechanisms responsible for these interstrain differences in transformation are unknown. However, the inherent quorum signaling may not be responsible since we induced the competence by exogenous addition of CSP18, thereby bypassing the need for the production and secretion of the CSP. Furthermore, the ComD and ComE proteins were also identical in both strains and were biologically active in GS‐5 (Biswas & Biswas, 2013).

It is well known that components of the Clp proteolytic complexes are involved in competence development in streptococci (Chastanet et al., 2001; Kajfasz et al., 2009, 2011; Lemos & Burne, 2002; Liu et al., 2017; Wahl et al., 2014; Weng et al., 2013). Several groups previously demonstrated that both ClpP and ClpX play a positive role in competence development in *S. mutans* when competence is induced at low cell density (OD_600_ = 0.15) (Kajfasz et al., 2011; Lemos & Burne, 2002). Our results also indicated that when the transforming DNA was added to low cell density, both ClpP and ClpX were required for optimum transformation. On the other hand, both ClpC and ClpE displayed an inhibitory effect on transformation at high cell density (Figure 5a). It has been previously shown in *S. mutans* and other streptococci that ClpC along with the MecA adaptor protein is involved in the degradation of the ComX sigma factor that is responsible for the expression of late competence genes (Biornstad & Havarstein, 2011; Dong et al., 2014; Son et al., 2018; Tian et al., 2013; Wahl et al., 2014). However, the role of ClpE in transformation has not been shown previously. It is not clear whether ClpE also plays a role in the degradation of the ComX protein in *S. mutans*. Surprisingly, we found that the effect of ClpP is opposite to ClpC. Since ClpC associates with ClpP protease to form a proteolytic complex, one would expect both ClpC and ClpP to have similar effects on transformation. It is possible that both ClpC and ClpE ATPases associate with some unknown protease to form a productive proteolytic complex that degrades ComX at low cell density; alternatively, the ATPase activity of ClpC and ClpE interferes with the transformation by some unknown mechanisms. Another explanation could be that during low‐cell density, some inhibitory peptides, which are substrates of ClpP, are present in the cell and interfere with the transformation. Several such inhibitory peptides have been identified in *S. mutans* and other streptococci (Ahn et al., 2014; Kaspar et al., 2015, 2018; Kim et al., 2013; Shields et al., 2018). The situation is somewhat different when the transforming DNA was added at a higher cell density (OD_600_ = 0.65). In this case, the inhibitory peptides are either absent or present in very low amounts. At this cell density, ClpC, ClpE, and ClpP all displayed negative effects on transformation perhaps because the ClpC/P and ClpE/P complexes are involved with the degradation of ComX and the other factors necessary for transformation. The role of ClpX in transformation is somewhat of a mystery since it is needed for transformation both at low‐ and high cell densities. It is noteworthy that, unlike ClpC and ClpE, ClpX expression is not under CtsR regulon and the level of ClpX remains constant throughout the growth (Gerth et al., 2004). Since ClpX forms complexes with ClpP, we speculate that the ClpX/P complex is responsible for the degradation of inhibitory peptides at both low and high cell densities. However, at high cell density, the effect of the inhibitory peptides is not as prominent as at the low cell density. *S. mutans* although does not encode the Lon protease, it does encode several other proteases such as SMU.2153 and PepO (Biswas et al., 2021; Underhill et al., 2019). ClpE negatively regulates SMU.2153 level in the cell; however, when we deleted SMU.2153, we did not find any noticeable change in the competence development [data not shown; Biswas et al., 2021]. On the other hand, PepO appears to negatively influence competence development only in defined or semidefined media (Underhill et al., 2019). Thus, we believe these two proteases do not play a role or are at least not directly involved in competence development under rich growth media. Further in‐depth studies are needed to understand the exact role of these Clp ATPases and ClpP in competence development in *S. mutans* UA159.

## AUTHOR CONTRIBUTIONS

**Satya D. Pandey**: Conceptualization (equal); data curation (equal); formal analysis (equal); investigation (equal); methodology (equal); validation (equal); visualization (equal); writing—original draft (equal); writing—review and editing (supporting). **Indranil Biswas**: Conceptualization (lead); data curation (equal); formal analysis (equal); funding acquisition (lead); investigation (equal); methodology (equal); project administration (lead); resources (equal); supervision (lead); validation (equal); visualization (equal); writing—original draft (supporting); writing—review and editing (lead).

## CONFLICTS OF INTEREST

None declared.

## ETHICS STATEMENT

None required.

## Data Availability

All data are provided in this article.

## 1

Figure A1.
